# Editorial: Receptor Biology and Cell Signaling in Diabetes

**DOI:** 10.3389/fphar.2022.864117

**Published:** 2022-03-16

**Authors:** Swayam Prakash Srivastava, Keizo Kanasaki

**Affiliations:** ^1^ Department of Pediatrics, Yale University School of Medicine, New Haven, CT, United States; ^2^ Vascular Biology and Therapeutics Program, Yale University School of Medicine, New Haven, CT, United States; ^3^ Internal Medicine 1, Shimane University Faculty of Medicine, Izumo, Japan

**Keywords:** endothelial cells, diabetes, fatty acid oxidation, glycolysis, mesenchymal gene expression, fibrosis, organ fibrosis, diabetic kidney disease

Diabetes mellitus affects almost half a billion people around the globe, and nearly 25% of diabetic people eventually develop proteinuria, vascular dysfunction, and related kidney disease (Cooper and Warren, 2019). Organ fibrosis among diabetic patients is a leading cause of death worldwide. The cellular mechanisms of organ fibrosis and the origin of these fibroblasts are poorly understood (Srivastava et al.). Diabetes linked receptor dysfunctions result in defective central metabolism, disrupted cytoskeleton, and compromised organelle function, and it is likely associated with activation of cellular transdifferentiation processes causing fibrogenesis in organs such as the kidneys, heart, and blood vessels, ultimately leading to organ failure (Kang et al., 2015; Srivastava et al., 2018). Myofibroblasts metabolic shifts, cytokine and chemokine reprogramming, and autophagy defects are key examples of a critical phenomenon that influence the destruction of cellular structures, deposition of extracellular matrix, and fibrogenic pathways.

Using contemporary cutting-edge technology, we have demonstrated the importance of glucocorticoid receptor (GR), fibroblast growth factor receptor 1, and sirtuin 3 mediated processes, which include suppression in myofibroblasts metabolic reprogramming, cytokine and chemokine reprogramming, inhibition in the levels of endothelial-to-mesenchymal transitions, and epithelial-to-mesenchymal processes in the diabetic kidneys (Figure 1) (Li et al., 2020a; Srivastava et al., 2021a; Srivastava et al., 2021c). Dipeptidyl transpeptidase-4 (DPP-4) and low-density lipoprotein receptor-related proteins 5 and 6 are key mesenchymal activators in diabetic tubules and endothelial cells. However, their interactions and their relation with myofibroblasts’ metabolic reprogramming is a matter of ongoing research. Improved understanding of the pathogenesis of diabetes and associated kidney disease is urgently needed to catalyze the development of new therapeutics. Available therapies are neither tissue- nor cell-specific and are ineffective in reversing kidney fibrosis and diabetic complications.

**FIGURE 1:**
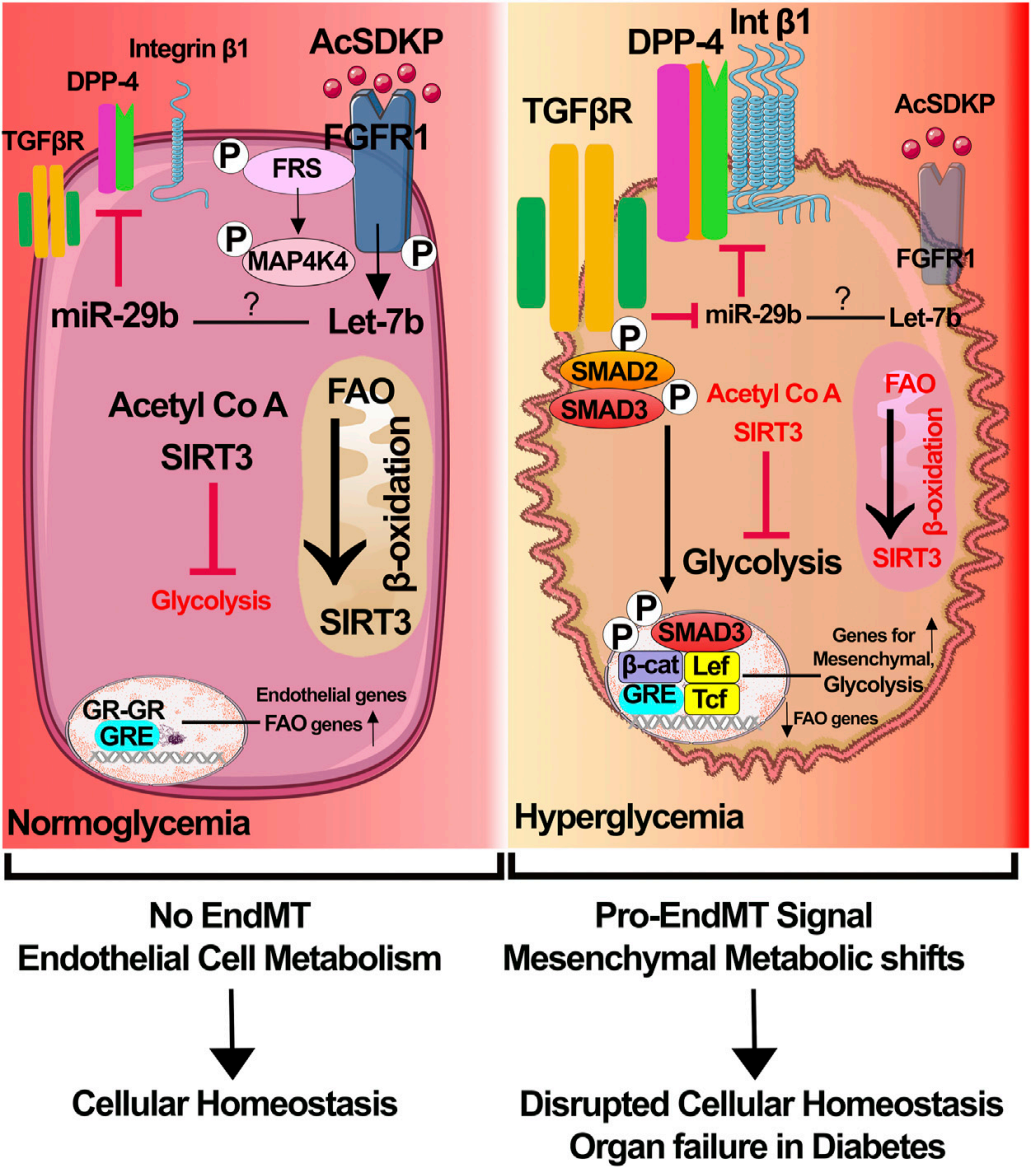
Receptor dysfunctions and metabolic myofibroblasts shifts in diabetic endothelial cells. In healthy endothelial cells, 1) physiological level of N-seryl-acetyl-lysyl-proline (AcSDKP)/fibroblasts growth factor receptor 1 (FGFR1) interaction leads to activation of signaling cascade that is important for expression of anti-mesenchymal microRNAs miR-29 and miR-let-7; 2) sirtuins 3 (SIRT3), which regulates the levels of fatty acid oxidation in mitochondria and glycolysis in cytosol, balances the endothelial cell metabolism; and 3) glucocorticoid receptor is key for the transcription of endothelial genes and fatty acid oxidation genes. Cumulative effects contribute to endothelial cell health and homeostasis. In diabetic endothelial cells, 1) suppression of AcSDKP/FGFR1 interaction causes downregulation of expression levels of miR-29 and miR-let-7, which leads to the elevation in the levels of mesenchymal transforming growth factor receptor 1 (TGFβR1), dipeptidyl peptidase-4 (DPP-4), and integrin β1; 2) SIRT3 deficiency causes suppression in the fatty acid oxidation and induction in the aberrant glycolysis; GR deficiency results in activation of Wnt signaling, endothelial-to-mesenchymal transition processes and suppression in the level of fatty acid oxidation. Accumulative effects of all these lead to myofibroblast metabolic reprogramming. Components of this figure were created using Servier Medical Art templates.

Recently, many renal protective agents that modulate the receptor physiology and improve cellular health and homeostasis, including sodium-glucose cotransporter inhibitors (SGLT-2), DPP-4 inhibitors, and N-seryl-acetyl-lysyl-proline, have been evaluated in preclinical settings and some in controlled clinical trials with promising outcomes (Kanasaki et al., 2014; Srivastava et al., 2016; Stavropoulos et al., 2018; Srivastava et al., 2020a; Srivastava et al., 2020b). Moreover, larger sample size is necessary to optimize their use in human beings.

In this Research Topic of Frontiers in Pharmacology, we discuss new pathophysiologic mechanisms related to receptor dysfunction and cell signaling in diverse cell types in diabetic kidneys, and targeting receptor dysfunction is one of the driving mechanisms in the development of future therapies against vascular dysfunction and fibrogenesis in diabetic kidney disease. In addition, we discuss the effect of modulators that inhibit the aberrant cell signaling in diabetic conditions. The knowledge gained from this issue will enhance the understanding of the complex nature of this disease which can be utilized to develop future therapeutics against diabetic kidney disease.

We focused on two sections.

## New Cellular Mechanisms

Podocytes are specialized differentiated cells, and loss of podocyte cells or podocyte foot process effacement are prominent features of diabetic nephropathy (Clement et al., 2011). GR and circulatory angiopoietin-like 4 proteins regulate podocyte-endothelium health and GR loss in podocytes leading to proteinuria and glomerulosclerosis in diabetes (Clement et al., 2014; Srivastava et al., 2021b). Epigenetic alterations, DNA methylation, and inability in DNA damage repair in diabetic podocytes are directly associated with diabetic nephropathy (DN) phenotypes. Vermon et al. study demonstrated the critical role of podocyte VEGF-A in the diffused glomerulosclerosis in diabetic and eNOS mutant mice. Deletion of VEGF-A in podocytes induced the abnormal S-nitrosylation of specific proteins, including β-integrin and laminin, which are involved in the development of diffused glomerulosclerosis, suggesting the essential role of VEGF-A and nitric oxide in the glomerular homeostasis and pathogenic roles of S-nitrosylated β-integrin and laminin in the glomerular disease, implicating the significant advances in podocyte cell biology (Vermon et al.). In addition, aberrant levels of ferroptosis, pyroptosis, necroptosis, and apoptosis are important phenomena in DN, linked with the activation of fibrogenic pathways in tubules and podocytes. Understanding gasdermin D mediated pyroptosis provides valuable insights into DN research. Another part of the study describes the role of mineralocorticoid receptor (MR) in diabetic kidney disease (DKD). MR antagonism reversed DKD phenotypes by suppressing fibrosis, oxidative stress, and inflammation (Kawanami et al.). Therefore, an enhanced understanding of MR biology in diverse cell types is important for DKD management.

## New Therapeutics

The use of SGLT2 inhibitors as safe therapeutics in diabetic kidney disease has recently been widely studied in preclinical settings and randomized clinical trials. The function of SGLT2 is to reabsorb urine glucose filtered from the glomerulus. The EMPA-REG trial has demonstrated that empagliflozin suppressed diabetic nephropathy phenotypes, and this study represents a crucial development in the clinical practice of diabetes management (Mayer et al., 2019). Data in mouse models suggest that SGLT2 inhibitors suppress the pathogenic abnormal glycolysis levels and restore the fatty acid oxidation levels in the tubules, improving tubular health (Li et al., 2020b).

One of the studies included in this issue imparts significant information on dapagliflozin in tubule protection. However, high salt diet intake compromised the tubular protection of dapagliflozin in diabetic patients and mouse models of DKD. These data demonstrate that high-salt diet intake impairs the tubule fatty acid metabolism, a key contributor of renal fibrosis in diabetes (Zou et al.). In addition, SGLT2 inhibitors are effective against autophagic defects in metabolic diseases. Autophagic improvement by SGLT2 inhibitors would be of great significance and usher the need for future randomized clinical trials to evaluate the SGLT2 inhibitors in autophagy defects related metabolic diseases. Zhang et al. tested the recombinant anti-IL6R fusion proteins in the mouse models of diabetic nephropathy and found that reducing DN phenotypes by mitigating the JAK2/STAT3 signal transduction pathway is effective, suggesting the pathobiology of IL-6R/JAK2/STAT3 pathway in DN (Zhang et al.). In recent years, ROCK inhibitors have been shown to effectively improve renal outcomes of diabetic patients suggesting the possibility of a potential lead in DKD.

## Conclusion

This Research Topic discussed the crucial pathways that will enlighten possible future therapeutics against fibrogenesis and vascular dysfunction in diabetes. The information gained from this Research Topic will be useful for clinicians and basic science researchers to guide novel therapeutic approaches and future research directions.
